# The Development of Diabetes after Subtotal Gastrectomy with Billroth II Anastomosis for Peptic Ulcer Disease

**DOI:** 10.1371/journal.pone.0167321

**Published:** 2016-11-28

**Authors:** Chien-Hua Chen, Che-Ming Hsu, Cheng-Li Lin, An-Kuo Chou, Long-Bin Jeng

**Affiliations:** 1 Digestive Disease Center, Show-Chwan Memorial Hospital, Changhua, Taiwan; 2 Hungkuang University, Taichung, Taiwan; 3 Chung Chou University of Science and Technology, Yuanlin Township, Changhua County, Taiwan; 4 Management Office for Health Data, China Medical University Hospital, Taichung, Taiwan; 5 College of Medicine, China Medical University, Taichung, Taiwan; 6 Department of Anesthesiology, China Medical University Hospital, Taichung, Taiwan; 7 School of Medicine, College of Medicine, China Medical University, Taichung, Taiwan; 8 Graduate Institute of Clinical Medical Science, School of Medicine, College of Medicine, China Medical University, Taichung, Taiwan; 9 Department of Surgery, Organ Transplantation Center, China Medical University Hospital, Taichung, Taiwan; University Hospital Oldenburg, GERMANY

## Abstract

**Purpose:**

A duodenal bypass after a Roux-en-Y gastric bypass operation for obesity can ameliorate the development of diabetes mellitus (DM). We attempted to determine the subsequent risk of developing DM after subtotal gastrectomy with Billroth II anastomosis (SGBIIA) for peptic ulcer disease (PUD).

**Methods:**

We identified 662 patients undergoing SGBIIA for PUD between 2000 and 2011 from the Longitudinal Health Insurance Database as the study cohort, and we randomly selected 2647 controls from the peptic ulcer population not undergoing SGBIIA and were frequency-matched by age, sex, and index year for the control cohort. All patient cases in both cohorts were followed until the end of 2011 to measure the incidence of DM. We analyzed DM risk by using a Cox proportional hazards regression model.

**Results:**

The patients who underwent SGBIIA demonstrated a lower cumulative incidence of DM compared with the control cohort (log-rank test, *P* < .001 and 6.73 vs 12.6 per 1000 person-y). The difference in the DM risk between patients with and without SGBIIA increased gradually with the follow-up duration. Age and sex did not affect the subsequent risk of developing DM, according to the multivariable Cox regression model. Nevertheless, the SGBIIA cohort exhibited a lower DM risk after we adjusted for the comorbidities of hypertension, hyperlipidemia, and coronary artery disease (adjusted hazard ratio (aHR): 0.56, 95% confidence interval (CI): 0.40–0.78). The incidence rate ratio (IRR) of DM in the SGBIIA cohort was lower than that in the control cohort for all age groups (age ≤ 49 y, IRR: 0.40, 95% CI: 0.16–0.99; age 50–64 y, IRR: 0.54, 95% CI: 0.31–0.96; age ≧ 65 y, IRR: 0.57, 95% CI: 0.36–0.91). Moreover, the IRR of DM was significantly lower in the SGBIIA cohort with comorbidities (IRR: 0.50, 95% CI: 0.31–0.78) compared with those without a comorbidity (IRR: 0.65, 95% CI: 0.40–1.04).

**Conclusion:**

The findings of this population-based cohort study revealed that SGBIIA was associated with a reduced risk of DM development, and the inverse association was greater in the presence of a comorbidity.

## Introduction

With the introduction of histamine-2 antagonists and proton pump inhibitors (PPIs) as well as the eradication of Helicobacter pylori (HP), the rate of elective operations for peptic ulcer disease (PUD) has been declining for the past 30 years [1]. However, surgery is still recommended for uncontrolled hemorrhage, gastrointestinal tract obstruction, or malignancy concerns [2]. A definite operation for PUD was considered unnecessary because of the introduction of PPIs and HP eradication after an improved understating of PUD pathogenesis [3]. Nevertheless, the location and extension of the ulcers responsible are the most critical determinants of the surgical method to be applied such as the decision to perform a gastrectomy for PUD. Resecting gastric ulcers located in a lesser curvature of the stomach is challenging because of the technical complexities induced by the rich blood arcades in the left gastric artery and obstruction tendencies caused by deformities of the gastric remnants after a wedge gastrectomy [4]. Regarding duodenal ulcers spanning more than 2 cm, the risk of leakage increases with a simple omental patch repair [5]. Therefore, a subtotal gastrectomy with Billroth II anastomosis (SGBIIA) is still recommended in targeting these PUDs.

The prevalence of diabetes mellitus (DM) is increasing rapidly worldwide, and type 2 DM constitutes more than 95% of DM-classified subgroups [6]. In 2010, the reported prevalence rates of DM in the adult populations of the United Kingdom, the United States, and mainland China were approximately 7%, 11%, and 15%, respectively [7–9]. The pathogenesis of type 2 DM includes insulin resistance and an insulin secretory response that is inadequate in fulfilling biological requirements. DM has imposed a substantial burden on socioeconomic status and public health because it is associated with the long-term damage of various organs such as the eyes, kidneys, the brain, and cardiovascular organs [10]. For example, the 5 most frequent complications of DM are amputation of the lower extremities, acute myocardial infarction, stroke, end-stage renal disease, and hyperglycemic-crisis-induced mortality [11].

A study reported that 85% of type 2 DM cases can result in remission after a Roux-en-Y bypass operation [12]. Duodenal diversion from contact with ingested nutrients, rather than weight loss, is reportedly a potential mechanism involved in the remission of type 2 DM [13]. Furthermore, SGBIIA is also a type of operation with a duodenal bypass because a gastrojejunostomy is performed after a gastrectomy.

In this study, we hypothesized that a history of SGBIIA for PUD treatment might be associated with a reduced risk of DM development. We conducted a nationwide population-based cohort study by analyzing data from a nationwide medical database, the National Health Insurance Research Database (NHIRD), to assess the association between SGBIIA and subsequent DM development.

## Methods

### Data Source

We used reimbursement claims data from the Longitudinal Health Insurance Database 2000 (LHID2000), established by the National Health Research Institutes (NHRI) under the Department of Health. LHID2000 contains all inpatient and outpatient medical claims for approximately 1 million people who were randomly sampled from the registry for beneficiaries of the NHIRD in the year 2000. The NHIRD is a nationwide database containing the claims records from Taiwan’s mandatory National Health Insurance (NHI) program, which was launched in 1995 and covers more than 99% of the population of Taiwan (23.74 million people) [14]. The NHRI have encrypted all patient identification numbers for privacy protection. The NHIRD comprises numerous registration data, and the details have been published in previous studies [15, 16]. Diagnoses were coded based on the International Classification of Diseases, Ninth Revision, Clinical Modification (ICD-9-CM). We obtained the data files (LHID2000) from the NHRI, which is a non-profit organization and the largest health research institute in Taiwan with many good statisticians and computer experts. The NHRI is responsible to provide appropriate data files to users. The distributions of sex, age and health care cost between the one million file (LHID2000) and the 23 million population were similar. The NHRI assigned a random number for each person by the method designed by Knuth, Park and Miller [17, 18].

### Data Availability Statement

All data and related metadata were deposited in an appropriate public repository in the National Health Research Institutes (NHRI). The data on the study population that were obtained from the NHIRD (http://nhird.nhri.org.tw/en/index.html) are maintained in the NHIRD (http://nhird.nhri.org.tw/). The NHRI is a nonprofit foundation established by the government. Only citizens of the Republic of China who fulfill the requirements of conducting research projects are eligible to apply for the NHIRD. The use of NHIRD is limited to research purposes only. Applicants must follow the Computer-Processed Personal Data Protection Law (http://www.winklerpartners.com/?p=987) and related regulations of National Health Insurance Administration and NHRI, and an agreement must be signed by the applicant and his/her supervisor upon application submission. All applications are reviewed for approval of data release.

### Ethics Statement

The NHIRD encrypts patient personal information to protect privacy and provides researchers with anonymous identification numbers associated with relevant claims information, including sex, date of birth, medical services received, and prescriptions. Therefore, patient consent is not required to access the NHIRD. This study was approved to fulfill the condition for exemption by the Institutional Review Board (IRB) of China Medical University (CMUH-104-REC2-115). The IRB also specifically waived the consent requirement.

### Sampled Participants

**Fig 1:**
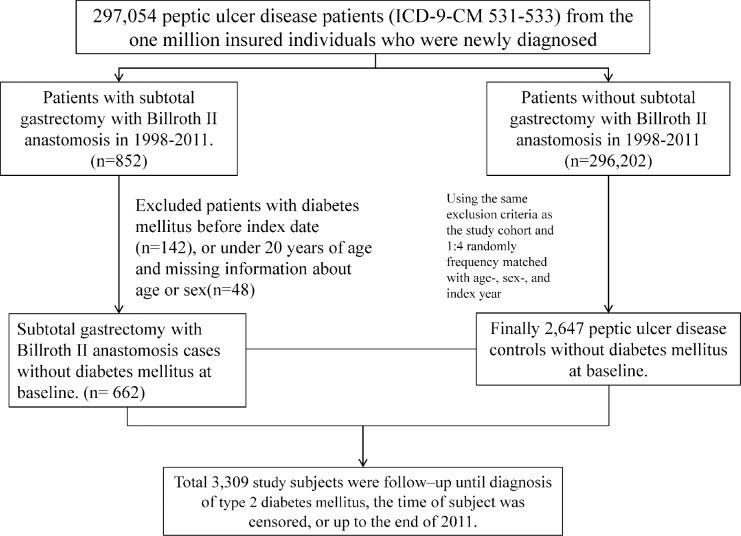
Participant selection process.

Fig 1 shows the participant selection process for the 2 study cohorts. All patients with a history of PUD (ICD-9-CM 531–533) between 1998 and 2010 were included in the study population. Patients who underwent SGBIIA (ICD-9-CM procedural code 43.7) were assigned to the SGBIIA cohort. Patients with a history of DM (ICD-9-CM 250), those younger than 20 years, and those with incomplete information were excluded. The date of SGBIIA was circumscribed as the index date. The non-SGBIIA cohort was established by randomly selecting the remaining patients who had PUD, but did not undergo SGBIIA. For each patient in the SGBIIA cohort, 4 non-SGBIIA patients without a history of diabetes were identified and frequency-matched by age (within a span of 5 y), sex, and the year of the index date. Baseline comorbidities included hypertension (ICD-9-CM 401–405), hyperlipidemia (ICD-9-CM 272), coronary artery disease (ICD-9-CM 410–414), stroke (ICD-9-CM 430–438), and chronic obstructive pulmonary disease (COPD) (ICD-9-CM 490,491, and 496).

### Outcome

The main outcome was based on the claims records with a diagnosis of type 2 DM (ICD-9-CM codes 250.x0 and 250.x2) during follow-up. The patient cases were followed from the index date to the date of type 2 DM diagnosis, the date of withdrawal from the insurance program, censorship because of death, or the end date of the database (ie, December 31, 2011).

### Statistical Analysis

We compared and examined the distributions of sex, age, and baseline comorbidities between the cohorts by using the Chi-square test to examine categorical variables and Student’s *t* test to analyze continuous variables. We assessed the cumulative incidences of type 2 DM between the SGBIIA and non-SGBIIA cohorts by using the Kaplan–Meier method, and we evaluated the differences by performing a log-rank test. The follow-up period (in person-years) was used to estimate the incidence density rates of type 2 DM. The incidence rate ratio (IRR) of the SGBIIA cohort to the non-SGBIIA cohort and the 95% confidence interval (CI) were estimated using a Poisson regression model. We also employed univariable and multivariable Cox proportional hazards regression models to assess the hazard ratio (HR) and 95% CI for type 2 DM. Variables in the multivariable model included sex, age, hypertension, hyperlipidemia, and coronary artery disease; all the confounding factors demonstrated a significant difference in the univariable Cox model. All analyses were performed using SAS software (version 9.4, SAS Institute Inc., North Carolina, USA); *P* < .05 indicated statistical significance.

## Results

**Fig 2:**
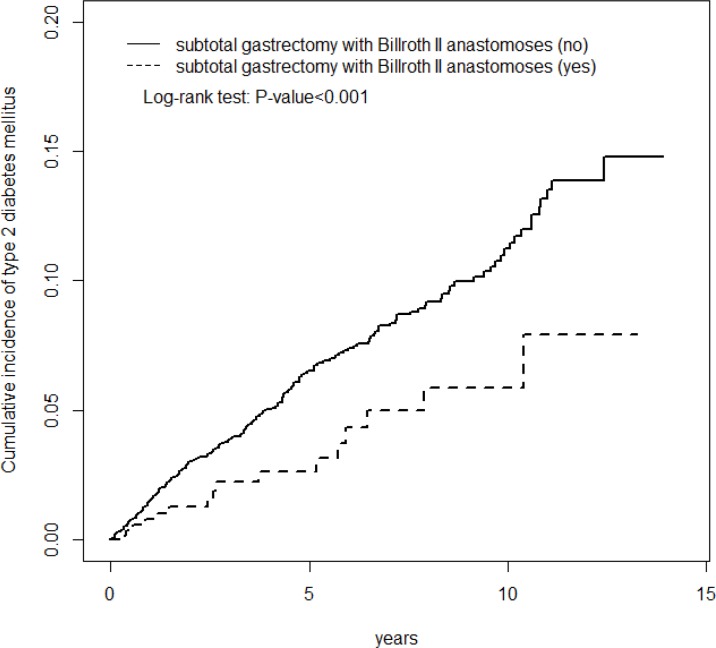
Cummulative incidence of type 2 DM in PUD patients with and without SGBIIA.

Fig 2 illustrates the cumulative type 2 DM incidence curves for the 2 cohorts, indicating that the incidence of DM in the SGBIIA cohort is significantly lower than that in the non-SGBIIA cohort (log-rank test, *P* < .001) during the mean follow-up period of 3.59 ± 3.61 (0.003–13.3) years for the SGBIIA cohort and 5.32 ± 3.71 (0.005–13.9) years for the non-SGBIIA cohort.

Table 1 lists the comparisons of the demographic characteristics and comorbidities between the PUD patients who underwent subtotal SGBIIA and those who did not. The SGBIIA cohort comprised 662 persons, whereas the non-SGBIIA cohort was composed of 2647 counterparts. In this study, 68.7% of the patients were men, and 54.7% of the patients were older than 65 years. The mean ages in the SGBIIA and non-SGBIIA cohorts were 65.0 years (SD = 14.5) and 64.7 years (SD = 14.5), respectively. Patients who did not undergo SGBIIA had a higher prevalence of comorbidities of hypertension, hyperlipidemia, coronary artery disease, and COPD (*P* < .05).

**Table 1:** Comparisons of demographic characteristics and comorbidities between PUD patients with and without SGBIIA

	Subtotal gastrectomy with Billroth II anastomosis		
	No	Yes	
	(N = 2647)	(N = 662)	*p*-value
**Gender**			0.99
Women	828(31.3)	207(31.3)	
Men	1819(68.7)	455(68.7)	
**Age stratified**			0.99
≤ 49	440(16.6)	110(16.6)	
50–64	760(28.7)	190(28.7)	
65+	1447(54.7)	362(54.7)	
Age, mean±SD ^a^	64.7(14.5)	65.0(14.5)	0.61
**Comorbidity**			
Hypertension	1383(52.3)	309(46.7)	0.01
Hyperlipidemia	650(24.6)	127(19.2)	0.004
Coronary artery disease	780(29.5)	158(23.9)	0.004
Stroke	207(7.82)	59(8.91)	0.36
COPD	637(24.1)	128(19.3)	0.01

Chi-square test

^a^ *t* test

Mean case group follow-up time = 3.59 (SD = 3.61)

Mean control group follow-up time = 5.32 (SD = 3.71)

Table 2 shows the results of the univariable and multivariable Cox proportional hazards regression models used for our analysis of the risk of variables contributing to type 2 DM. The risk of developing type 2 DM was greater for patients with the comorbidities of hypertension (adjusted HR (aHR) = 1.34, 95% CI = 1.09–1.64) and hyperlipidemia (aHR = 1.50, 95% CI = 1.22–1.85).

**Table 2:** HRs of type 2 DM in association with age, sex, and comorbidities in univariable and multivariable Cox regression models

	Crude^&^		Adjusted^†^	
Variable	HR	(95%CI)	HR	(95%CI)
**Subtotal gastrectomy with Billroth II anastomosis**	0.53	(0.38,0.74)***	0.56	(0.40,0.78)***
**Age, years**	1.00	(1.00, 1.01)	-	-
**Sex(female vs male)**	0.90	(0.74, 1.09)	-	-
**Baseline co-morbidities (yes vs no)**				
Hypertension	1.50	(1.25,1.80)***	1.34	(1.09,1.64)***
Hyperlipidemia	1.66	(1.37,2.03)***	1.50	(1.22,1.85)***
Coronary artery disease	1.27	(1.04, 1.55)*	1.00	(0.81,1.24)
Stroke	0.81	(0.52, 1.25)	-	-
COPD	1.01	(0.80, 1.27)	-	-

Crude HR^**&**^ represents the relative hazard ratio; Adjusted HR^†^ represents adjusted hazard ratio: mutually adjusted for comorbidities of hypertension, hyperlipidemia, and coronary artery disease in the Cox proportional hazard regression models.

*P < .05

***P < .001

Table 3 lists a comparison of the incidence densities of the type 2 DM HRs between PUD patients who underwent SGBIIA versus those who did not after they were stratified by demographic characteristics and comorbidities. During the 12-year follow-up, we noted a lower incidence density rate of type 2 DM in the SGBIIA cohort compared with the non-SGBIIA cohort (6.73 and 12.6, respectively, per 1000 person-y), with an IRR of 0.53 (95% CI = 0.38–0.74). The IRR of type 2 DM in the SGBIIA cohort was lower than that in the non-SGBIIA cohort for both sexes. Compared with the non-SGBIIA cohort, the SGBIIA cohort was associated with a lower risk of type 2 DM, irrespective of the age group. A greater incidence density rate of type 2 DM was found in patients with comorbidity in both groups. However, the patients who underwent SGBIIA were at a substantially lower risk of type 2 DM compared with those who did not when they had a comorbidity (IRR = 0.50, 95% CI = 0.31–0.78). Notably, the risk of developing type 2 DM did not increase with increasing age in Table 2. Moreover, Table 3 shows that the rate of developing type 2 DM peaked in individuals with age 50–64 years rather than in the elderly (greater than 65 years).

**Table 3:** Comparison of incidence densities of type 2 DM HR between PUD patients with and without SGBIIA stratified by demographic characteristics and comorbidities

	Subtotal gastrectomy with Billroth II anastomosis						
	No			Yes			
	Event	PY	Rate^#^	Event	PY	Rate^#^	IRR^&^ (95% CI)
**All**	178	14101	12.6	16	2376	6.73	0.53(0.38, 0.74)***
**Gender**							
Women	62	4426	14.0	4	788	5.08	0.36(0.19, 0.71)**
Men	116	9675	12.0	12	1588	7.56	0.63(0.43, 0.93)*
**Stratify age**							
≤ 49	28	2871	9.75	2	517	3.87	0.40(0.16, 0.99)*
50–64	72	4173	17.3	7	750	9.33	0.54(0.31, 0.96)*
65+	78	7056	11.1	7	1108	6.32	0.57(0.36, 0.91)*
Comorbidity^‡^							
No	50	5061	9.88	7	1092	6.41	0.65(0.40, 1.04)
Yes	128	9039	14.2	9	1283	7.01	0.50(0.31, 0.78)**

Rate^#^, incidence rate, per 1000 person-years; IRR^&^, incidence rate ratio

Comorbidity^‡^: Patients with any of the comorbidities of hypertension, hyperlipidemia, coronary artery disease, stroke, and COPD were classified as the comorbidity group

*P < .05

**P < .01

***P < .001

## Discussion

Table 4 shows that the incidence of PUD decreased progressively in our study population, which might be explained by improving living conditions with lower HP infection and the introduction of PPIs. The incidence in our study was similar to those of several former reports in Taiwan with incidence of 9.52% per 1000 person-years in a 2001–2009 cohort study based on LHID 2000 and prevalence of 9.4% in a cross-sectional study of individuals for self-paid physical check-up conducted in 2008 [19, 20]. In 1 million people, 297054 patients received a diagnosis of PUD; therefore, the prevalence was approximately 29.7% in our study. Moreover, 0.29% (852 of 297054) of the patients with PUD would undergo SGBIIA in our study. In accordance with the literature, our findings revealed that SGBIIA, which might be indicative of the presence of complicated PUD, was performed mostly in male patients and those older than 65 years. The most common reasons for the propensity of older adults to incur complicated PUD may include a greater prevalence in the HP infection rate and the associated poor mucosal resistance for older patients, a linear increase in non-steroidal anti-inflammatory drug (NSAID) usage with age, and a greater prevalence of smoking habits in the older population [21–24]. With the exception of an HP infection, unrelated to the risk of perforation, all the mentioned risk factors are associated with the risks of hemorrhage and perforation in patients with PUD. Moreover, the prevalence rate of HP infection, NSAID usage, and smoking habits is higher in men.

**Table 4:** Annual incidence of PUD in Taiwan population

year	Total population	PUD	IR
1998	638080	10240	16.05
1999	653852	23627	36.14
2000	668657	23150	34.62
2001	673461	18032	26.78
2002	677752	15553	22.95
2003	683220	12519	18.32
2004	688815	11838	17.19
2005	697144	9533	13.67
2006	703958	8295	11.78
2007	710048	7672	10.8
2008	716391	6781	9.47
2009	721504	6376	8.84
2010	727666	5690	7.82
2011	732446	4264	5.82

IR: Incidence rate per 1,000

Our study findings implied that hypertension and hyperlipidemia were associated with an increased risk of DM development. In murine and human β cells, a study revealed that low-density lipoproteins could lower both the proliferation of β cells and maximal insulin secretion after glucose stimulation [25]. By contrast, another study reported that high-density lipoproteins could prevent basal and interleukin-1 or glucose-stimulated β-cell apoptosis. Moreover, the elevated levels of free fatty acids are a critical determinant of insulin resistance [26]. Hypertension and DM are a negative combination [27]. Hypertension can activate the renin-angiotensin-aldosterone system (RAAS), and they influence each other. Through RAAS activation, inflammation and oxidative stress are induced to increase insulin resistance [28]. Moreover, insulin resistance can result in vascular smooth muscle proliferation and arterial stiffness, increase vascular tone, and reduce vasodilation, leading to hypertension [29].

The inverse association between SGBIIA and DM is attributable to a greater prevalence of critical DM risk factors in patients who did not undergo SGBIIA compared with those who did. Moreover, the mean-follow-up period was shorter in patients who underwent SGBIIA than that in those without undergoing SGBIIA. However, one can reasonably conclude that the reduced risk of DM observed in the SGBIIA patients was more likely a consequential effect of the SGBIIA status because the possible confounding effect of DM risk factors was minimized substantially in our study (Table 2). We observed a consistent drop in the DM risk post-SGBIIA in both sexes and for all age groups. Furthermore, the inverse association between SGBIIA and DM was greater in the presence of a comorbidity because the IRR of DM was significantly lower in the SGBIIA patients with a comorbidity compared with those without (Table 3). In addition, our findings also consistently revealed that the inverse association increased progressively over the whole 12-year-long follow-up duration despite that the mean-follow-up period was shorter in patients who underwent SGBIIA than that in those without undergoing SGBIIA (Fig 2). These findings, combined with the results of the subgroup analyses confirm the possible inverse association between SGBIIA and DM. However, it requires more studies to clarify whether it is epiphenomenon or causal relationship.

Several studies have suggested that the inverse association between SGBIIA and DM may be due to the following 4 mechanisms. First, starvation or a reduced fat mass after a gastrectomy leads to lower lipid oxidation and enhanced insulin sensitivity [30]. Moreover, insulin secretion can be enhanced after weight loss [31]. Second, ghrelin can increase the hypothalamic expression of neuropeptide Y to stimulate appetite and lessen insulin secretion. However, the level of ghrelin, which is secreted chiefly by oxyntic cells in the stomach, decreases after a gastrectomy [32]. Third, the proximal intestines contribute to the regulation of the glucose metabolism, as suggested by foregut theory [33]. The direct passage of nutrients to the intestinal foregut with a stomach bypass triggers the counter-regulation of anti-secretin, which delays the insulin response and impairs insulin action. Finally, the distal intestines can regulate the secretion of the gut hormone, as suggested by hindgut theory, to maintain the glucose metabolism in a state of homeostasis [34, 35]. The rapid transit of undigested nutrients to the distal intestines upregulates the production of glucagon-like peptide 1 (GLP-1) and peptide-YY; moreover, GLP-1 can stimulate insulin secretion and antagonizes β-cell apoptosis, whereas peptide-YY can reduce insulin resistance. Therefore, the mentioned mechanisms interact with each other, and others cannot necessarily be precluded [36].

To the best of our knowledge, this is the first population-based study to investigate whether SGBIIA for PUD might be inversely associated with a reduced risk of DM development. We adopted a longitudinal design, rather than a cross-sectional approach, to evaluate this association. The statistical analysis results were strengthened through our use of the national database, which contained the relevant medical data of a representative cohort of 1 million people over a 12-year observation period. Furthermore, the sampled patients were from a stable population of Taiwan participating in the NHI program, which includes the records of approximately 99% of the population.

Our study had several limitations. First, we may have overlooked potential confounding factors because the NHIRD does not provide detailed information regarding the DM-related lifestyle, socioeconomic status, and the family history of patients. However, we controlled for potential DM-associated comorbidities in our study, and SGBIIA was found to be inversely associated with the development of DM. Second, patients who were not covered by the NHI program could not be included in our study population. Nevertheless, this program currently covers more than 99% of the population of Taiwan. Finally, it is difficult to clearly depict the precise mechanism for the protective effect of SGBIIA against DM. However, the mentioned 4 possible mechanisms always interact with each other and cannot operate independently. Furthermore, an inverse association between SGBIIA and ischemic stroke, rather than hemorrhagic stroke, was also consistently demonstrated in our former publication [37].

In conclusion, this population-based cohort study revealed that SGBIIA was associated with a reduced risk of DM development, and the inverse association was greater in the presence of a comorbidity.

## Abbreviations

DM: diabetes mellitus
SGBIIA: subtotal gastrectomy with Billroth II anastomosis
PUD: peptic ulcer disease
aHR: adjusted hazard ratio
CI: confidence interval
IRR: incidence rate ratio
PPIs: proton pump inhibitors
HP: Helicobacter pylori
NHIRD: National Health Insurance Research Database
LHID 2000: Longitudinal Health Insurance Database 2000
NHRI: National Health Research Institute
NHI: National Health Insurance
ICD-9-CM: International Codes of Disease 9^th^ Edition Clinical Modification
COPD: chronic obstructive pulmonary disease
NSAIDs: non-steroidal anti-inflammatory drugs
LDL: low-density lipoproteins
HDL: high-density lipoproteins
RAAS: renin-angiotensin-aldosterone system
GLP-1: glucagon-like peptide 1
